# Supplementary material for: Enhancing the dipolar coupling of a S-T0 qubit with a transverse sweet spot
Source: Nat Commun. 2019 Dec 10;10:5641. doi: 10.1038/s41467-019-13548-w (PMC6904552; doi:10.1038/s41467-019-13548-w)
Supplement: Supplementary file 1 — Supplementary Information [file 41467_2019_13548_MOESM1_ESM.pdf]

Supplementary Information for "Enhancing the dipolar coupling of a S-T_0 qubit with a transverse sweet spot" by Abadillo-Uriel et al.

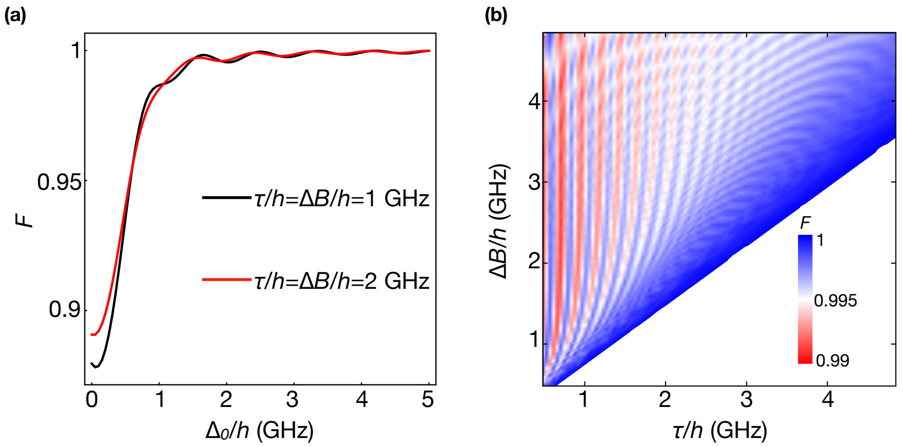

Supplementary Fig. 1. AC-driven single-qubit X_π gate fidelities in the absence of charge noise. **a** Gate fidelity F as a function of qubit-resonator detuning Δ_0 for two different values of τ and ΔB (and hence, ω_q). Far off resonance, no photons are excited in the cavity (i.e., no leakage), and the gate fidelity approaches unity. Near the resonance condition $\Delta_0 = 0$, photonic leakage is enhanced. (Note that the fidelity axis does not extend all the way to $F = 0$ here, for visual clarity.) **b** Gate fidelity F as a function of tunnel coupling τ and magnetic field gradient ΔB at the TSS, for a fixed value of $\Delta_0/h = 1.5$ GHz. As explained in the main text, the oscillations here can only be attributed to strong-driving effects.

Supplementary Note 1. CHARACTERIZING THE SOP SWEET SPOT

In the main text, we discuss the order of the sweet spot at the symmetric operating point (SOP). This requires evaluating the qubit frequency ω_q and its derivatives $d^n\omega_q/d\varepsilon^n$ at the sweet spot. To simplify the derivation, ω_q can be expanded in terms of the small parameters ε/U, τ/U, and $\Delta B/U$. Here we explain how the calculations are performed using Mathematica symbolic manipulation software[1]. Since the resulting analytical expressions are complicated, we outline the steps of the calculations here, and provide details in the accompanying Supplemental Mathematica Notebook.

First, we divide the singlet-triplet Hamiltonian $H_{\rm ST}$ [Eq. (1) of the main text] by U, and express all quantities in terms of the dimensionless parameters ε/U, τ/U, and $\Delta B/U$. $H_{\rm ST}$ is then diagonalized. The two lowest energy eigenvalues E_0 and E_1 are identified by evaluating the symbolic expressions at typical values of ε, τ, ΔB, and U. This allows us to construct a formal expression for $\hbar\omega_q = E_1 - E_0$.

Second, we expand $f_q = \omega_q/2\pi$ in terms of the small parameters ε/U, τ/U, and $\Delta B/U$. The resulting series takes the form

$$hf_q(\varepsilon,\tau,\Delta B,U) = U\sum_{i=0} f_i(\tau/U,\Delta B/U)(\varepsilon/U)^i, \tag{1}$$

and is expected to converge rapidly for realistic parameter values.

Third, we solve for several f_i by taking appropriate derivatives of ω_q. Due to the symmetry of the SOP, we note that the odd-order derivatives, $d^{2n-1}\omega_q/d\varepsilon^{2n-1}|_{\rm SOP}$, all vanish for $n \geq 1$, yielding $f_{2n-1} = 0$. We also note that the term $f_2(\tau/U, \Delta B/U)$ vanishes when the dispersion curvature changes sign at the super sweet spot $\tau = \tau_{\rm SSS}$. Solving $f_2(\tau_{\rm SSS}/U, \Delta B/U) = 0$ then gives $\tau_{\rm SSS}/U = \sqrt{3/2}\,\Delta B/U + \mathcal{O}\left[(\Delta B/U)^5\right]$, as reported in the main text.

Finally, after setting $\tau_{\rm SSS} = \sqrt{3/2}\,\Delta B$, we can evaluate several terms in Eq. (1), obtaining

$$\begin{aligned} f_4(\tau_{\rm SSS}/U,\Delta B/U) &= 0 + \mathcal{O}\left[(\Delta B/U)^3\right], \\ f_6(\tau_{\rm SSS}/U,\Delta B/U) &= 0 + \mathcal{O}\left[(\Delta B/U)^3\right], \\ f_8(\tau_{\rm SSS}/U,\Delta B/U) &= 0 + \mathcal{O}\left[(\Delta B/U)^3\right], \\ f_{10}(\tau_{\rm SSS}/U,\Delta B/U) &= 0 + \mathcal{O}\left[(\Delta B/U)^3\right]. \end{aligned}$$

We note, however, that even though $(\Delta B/U)^3 \ll 1$, the tenth order term f_{10} has a large prefactor, such that $f_{10}(\tau_{\rm SSS}/U, \Delta B/U) > 0.1$ for typical parameter values. Hence, we refer to the SOP with $\tau = \tau_{\rm SSS}$ as an approximate ninth-order sweet spot, which explains the extreme flatness of the curvature.

Supplementary Note 2. OTHER NOISE SOURCES

Although detuning fluctuations dominate the noise over a large portion of the parameter space, other noise mechanisms may be relevant in some cases. In this Supplemental Section we consider two other noise sources: tunnel-coupling noise and Rabi-frequency fluctuations.

A. Tunnel-coupling noise

In general, this type of noise is expected to be much weaker than detuning noise. However, it can become the leading decoherence mechanism in some cases, such as at the SOP[2], which is a sweet spot for the detuning. One challenge for treating this mechanism theoretically is the dearth of experimental studies. To estimate the noise amplitude, we therefore follow the theoretical analysis of ref 3, where the tunnel-coupling noise is related to the detuning noise, using WKB theory to estimate the tunnel coupling. This analysis suggest that $c_\tau \approx 3.2 \times 10^{-4} c_\varepsilon$, where c_τ and c_ε are the amplitudes of the $1/f$ noise distributions of tunnel-coupling and detuning fluctuations, as defined in Eq. (6) of the main text. With this result, we estimate the decoherence time associated with tunnel-coupling noise using the same simulation method that we previously applied to detuning noise. For details of the simulation, see Supplementary Note 6, where we specifically consider the SOP operating point. For the TSS operating point, our results are shown in Supplementary Fig. 2(a) where we obtain coherence times in the range of 1-6 μs. For single-qubit gates, tunnel-couple noise has a negligible effect. However, two-qubit gates are somewhat slower, such that the effects of tunnel-coupling noise are comparable to detuning noise.

B. Rabi-frequency fluctuations

When the qubit is driven, to perform single-qubit gate operations, it is susceptible to fluctuations of the detuning parameter that directly affect the Rabi frequency[4]. Since leakage was already considered in the main text, we ignore it here, allowing us to obtain a simple analytical estimate. Eliminating the leakage state, the Hamiltonian describing Rabi oscillations is given by

$$H_{\text{Rabi}} = \frac{\omega_0}{2}\sigma_z + d_{01}(\varepsilon)\varepsilon_{\text{AC}}\cos(\omega_0 t)\sigma_x. \tag{2}$$

After moving to the rotating frame defined by $H_0 = \frac{\omega_0}{2}\sigma_z$ and applying a rotating-wave approximation, the Hamiltonian reduces to

$$H'_{\text{Rabi}} = d_{01}\frac{\varepsilon_{\text{AC}}}{2}\sigma_x. \tag{3}$$

An additional rotation about the y axis gives

$$H''_{\text{Rabi}} = d_{01}\frac{\varepsilon_{\text{AC}}}{2}\sigma_z. \tag{4}$$

Now, assuming the detuning fluctuations are quasistatic, taken from a Gaussian distribution with standard deviation $\sigma_\varepsilon = 2\ \mu$eV, we can perform an analytic average over the time evolutions of the density matrix associated with H''_{Rabi}, obtaining the dephasing time $T_\varphi^R = 1/\Gamma_\varphi^R$, where

$$\Gamma_\varphi^R = \frac{\varepsilon_{\text{AC}}}{4\hbar}\frac{d(d_{01})}{d\varepsilon}\sigma_\varepsilon. \tag{5}$$

Supplementary Figure 2(b) shows our analytical estimate for T_φ^R, obtained from Eq. (5). As can be seen, the effect of Rabi-frequency fluctuations is generally weaker than leakage, as plotted in Fig. 3(c) of the main text. However, we observe clear similarities between T_φ^R and T_L. To explain this, we note that Rabi-frequency fluctuations arise directly from d_{01} in the system Hamiltonian [see Eq. (5)], rather than its prefactor [compare Eq. (4) of the main text]. The dynamics leading to T_φ^R are not caused by leakage; however since the charge dipole, d_{01}, arises from the hybridization of logical and leakage states, Rabi-frequency fluctuations and fluctuation-induced leakage cause similar dephasing behavior of the logical states.

In the frame of H''_{Rabi}, the Lindblad dephasing operator is given by $\frac{\Gamma_\varphi^R}{2}(\sigma_z\rho''\sigma_z)$. However, the master equation simulations, described in Methods, are performed in the lab frame. Transforming back to this frame, the Lindblad

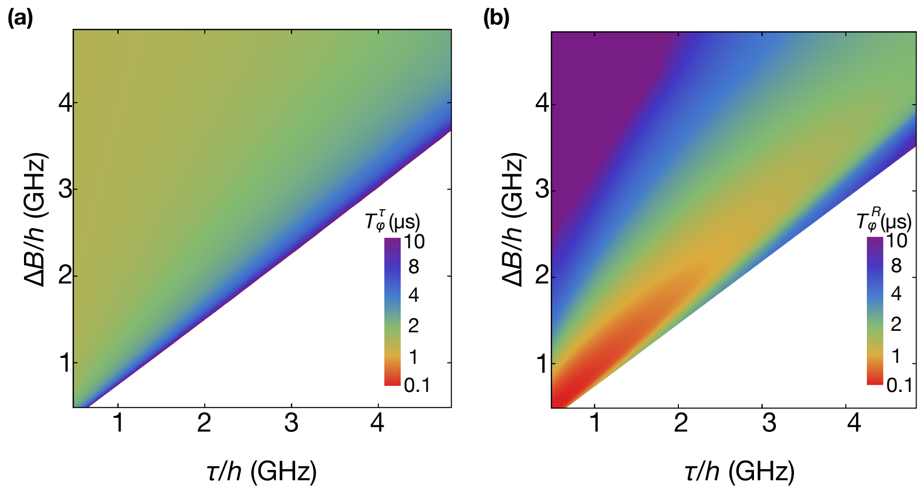

Supplementary Fig. 2. Dephasing times due to non-leading noise sources, plotted with the same color scale as Figs. 3(b,c) of the main text. (a) Dephasing time T_φ^τ, obtained from simulations with $1/f$-type tunnel-coupling noise. (b) Analytical estimate of the dephasing time T_φ^R, caused by Rabi-frequency fluctuations.

operator becomes $\frac{\Gamma_\varphi^R}{2}(\sigma^+\rho\sigma^- + \sigma^-\rho\sigma^+ - \rho)$. We incorporate this dephasing term into the simulations of single-qubit gates, using Γ_φ^R from Eq. (5). The effects of Rabi-frequency fluctuations are included in Fig. 3(d) of the main text (as are all other decoherence mechanisms considered in this work). Since these single-qubit gate operations are performed in the strong-driving regime, Rabi-frequency fluctuations here become the dominant decoherence mechanism. However, for two-qubit gates, where $g_0 \ll \varepsilon_{\mathrm{AC}}$, fluctuations of g_0 do not significantly affect gate the fidelities; therefore, we do not include them in the two-qubit master equation simulations reported in Fig. 4(a) of the main text.

C. Wavefunction displacement

The presence of detuning noise induces random displacement of the electron wavefunction which, in turn, changes the gradient that affects the qubit. Following the Supplemental Material from Ref. 5, and neglecting valley-orbit interactions that would strongly depend on interfacial disorder, this displacement is expected to be ±4 pm for a nearby two-level fluctuator inducing the noise. For a gradient on the order of 1 mT·nm^{-1} (which corresponds to 100 mT for dots separated by about 100 nm), this corresponds to ±4 μT fluctuations.

In this way, we can estimate a lower bound for the coherence time for this mechanism for the case of a pure spin-qubit. A 4 μT fluctuation is also the expected fluctuation for a spin-qubit in 99.99% purified ^{28}Si, corresponding to a decoherence time of 5 μs (see Ref. 6). At the TSS, however, the spin degree of freedom hybridizes with the charge degree of freedom. Hence, compared to the previously considered mechanisms, this decoherence mechanism should be negligible.

Supplementary Note 3. SINGLE-QUBIT FIDELITY OSCILLATIONS

In Fig. 3d of the main text we observe weak oscillations of the single-qubit gate fidelity very near the resonance condition, which is indicated as a dashed line. Here we show that this behavior occurs due to a combination of leakage and strong driving effects.

The full Hamiltonian of the qubit-cavity system is given in Eq. (4) of the main text as

$$H_{qr} = \hbar\omega_r a^\dagger a + \sum_n E_n \sigma_{nn} + \sum_{n,m} \left[\Delta\varepsilon d_{nm}\sigma_{nm} + \hbar g_0 d_{nm}(a + a^\dagger)\sigma_{nm}\right], \tag{6}$$

where all parameters are described in the main text. In the following discussion, we can ignore the leakage state L in

this expression, since it plays no important role. Evaluated at the TSS, Eq. (6) then reduces to

$$H_{qr}^{(0,1)} = \frac{\hbar\omega_q}{2}\sigma_z + \hbar\omega_r a^\dagger a + \varepsilon_{\rm AC} d_{01}\cos(\omega_q t)\sigma_x + \hbar g(a + a^\dagger)\sigma_x, \tag{7}$$

where the resonant drive is given by $\Delta\varepsilon = \varepsilon_{\rm AC}\cos(\omega_q t)$, and $g = g_0 d_{01}$. We can gain insight into the fidelity oscillations of Fig. 3d by moving to the rotating frame defined by

$$H_0 = \frac{\hbar\omega_q}{2}\sigma_z + \hbar\omega_r a^\dagger a. \tag{8}$$

The resulting interaction Hamiltonian is given by

$$H_{\rm int} = \frac{\varepsilon_{\rm AC}}{2} d_{01}\sigma_x + \hbar g(a^\dagger\sigma_- e^{i\Delta_0 t/\hbar} + a\sigma_+ e^{-i\Delta_0 t/\hbar}) + \hbar g(a^\dagger\sigma_+ e^{i(\omega_q+\omega_r)t/\hbar} + a\sigma_- e^{-i(\omega_q+\omega_r)t/\hbar}). \tag{9}$$

Here, the first term is responsible for Rabi oscillations. The second term causes unwanted excitations of photons in the cavity, corresponding to leakage, and its effect is maximized at the resonance condition, $\Delta_0 = 0$. The third term contains the counter-rotating terms; however both the second and third terms correspond to strong-driving effects.

In Supplementary Fig. 1a, we plot the fidelity of an X_π gate as a function of the qubit-cavity detuning Δ_0. (Note that since τ and ΔB are fixed for each curve, this corresponds to varying the parameter ω_r.) Here, we do not include charge noise, so the main features in the plot are caused by leakage. However, a smaller contribution can also be ascribed to the counter-rotating terms. To demonstrate strong-driving effects more vividly, in Supplementary Fig. S1b we plot the fidelity of single-qubit gate operations at a TSS, as function of ΔB and τ, while holding Δ_0 fixed. (Again, this requires varying ω_r. This result differs from Fig. 3d in the main text, where ω_r is held fixed.) Since the X_π gate time ($t = t_g$) depends only on $\varepsilon_{\rm AC} d_{01}$, which is a non-oscillating, monotonic function of τ and ΔB, the presence of oscillations in this figure can only be attributed to strong driving.

Supplementary Note 4. TWO-QUBIT GATE FIDELITY OSCILLATIONS

The qubit decay rate is known to be enhanced when the qubit and cavity are resonant, due to the Purcell effect[7]. Detuning the qubit(s) from the cavity suppresses this effect. In particular, in the dispersive regime, the Purcell decay is given asymptotically by $\gamma_P \approx (\hbar g/\Delta_0)^2\kappa$. In Fig. 4a of the main text, we observe this type of behavior near the resonance condition. Further away from the resonance condition, the fidelity quickly reaches its maximum value and begins to decrease again. In the main text, we explain this behavior as arising from the cumulative effects of dephasing, as the gate time becomes very long when the detuning Δ_0 is large. In addition to these general trends, Fig. 4a also exhibits small, fast oscillations very near the resonance condition. In Supplementary Fig. 2, we show a blown-up view of those results, for the same device parameters. We now show that the oscillations are caused by a combination of leakage and strong driving.

The full Hamiltonian of the qubit-cavity-qubit system is given in Eq. (5) of the main text as

$$H_{qqr} = \hbar\omega_r a^\dagger a + \sum_{i=a,b}\sum_n E_{n,i}\sigma_{nn,i} + \sum_{i=a,b}\sum_{n,m}\hbar g_{0,i} d_{nm,i}(a + a^\dagger)\sigma_{nm,i}, \tag{10}$$

After eliminating the leakage states, as in the previous section, and moving to the rotating frame defined by

$$H_0 = \frac{\hbar\omega_{q,a}}{2}\sigma_{z,a} + \frac{\hbar\omega_{q,b}}{2}\sigma_{z,b} + \hbar\omega_r a^\dagger a, \tag{11}$$

Eq. (10) reduces to

$$H_{\rm int} = \sum_{i=a,b}\left[\hbar g_i(a^\dagger\sigma_{-,i} e^{i\Delta_0 t/\hbar} + a\sigma_{+,i} e^{-i\Delta_0 t/\hbar}) + \hbar g_i(a^\dagger\sigma_{+,i} e^{i(\omega_{q,i}+\omega_r)t/\hbar} + a\sigma_{-,i} - e^{-i(\omega_{q,i}+\omega_r)t/\hbar})\right], \tag{12}$$

where $g_i = g_{0,i} d_{01,i}$ Here, the first term is responsible for the effective iSWAP interaction (after eliminating the photon states via a canonical transformation). It can also cause leakage by exciting photons in the cavity, as well as the Purcell effect, when $\kappa > 0$. The second term contains counter-rotating (i.e., strong driving) terms and is responsible for the fast oscillations observed in Supplementary Fig. 2. It is interesting to note that, for the case of a high quality factor (e.g., $Q = 10^5$ used in this simulation), the effect of the counter-rotating terms can actually dominate over the Purcell decay, allowing the fidelity to recover its high value in very narrow bands, as shown in the figure. However exploiting this effect would require exceptionally fine tuning of the device.

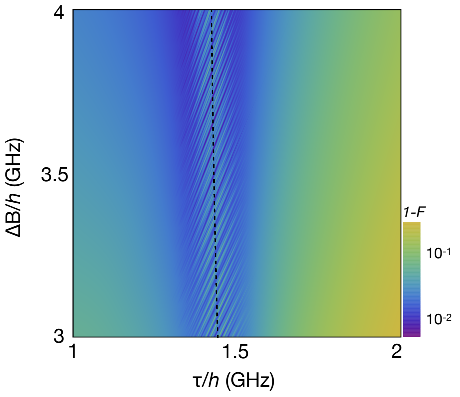

Supplementary Fig. 3. A blown-up view of the results in Fig. 4a of the main text, using all the same simulation parameters. The dashed black line marks the resonance condition.

Supplementary Note 5. INCLUDING A GLOBAL B-FIELD AND TRANSVERSE FIELD GRADIENTS

In Fig. 4b of the main text, we explore the two-qubit iSWAP gate errors caused by leakage into the spin-polarized states. In this section, we provide details of that calculation.

The two-qubit iSWAP gate fidelity is computed in two steps, following the same procedure used for simulations with just the spin-non-polarized states. First, we expand the singlet-triplet Hamiltonian in Eq. (1) of the main text to include the polarized triplet states, $|T_-\rangle = |\uparrow\uparrow\rangle$ and $|T_+\rangle = |\downarrow\downarrow\rangle$.

For the basis set $\{|S(1,1)\rangle, |T_0(1,1)\rangle, |S(0,2)\rangle, |S(2,0)\rangle, |T_-(1,1)\rangle, |T_+(1,1)\rangle\}$ we then obtain

$$H_{\rm ST} = \begin{pmatrix} 0 & \Delta B & \sqrt{2}\tau & \sqrt{2}\tau & -\frac{\Delta B_\perp}{\sqrt{2}} & \frac{\Delta B_\perp}{\sqrt{2}} \\ \Delta B & 0 & 0 & 0 & 0 & 0 \\ \sqrt{2}\tau & 0 & -\varepsilon & 0 & 0 & 0 \\ \sqrt{2}\tau & 0 & 0 & 2U+\varepsilon & 0 & 0 \\ -\frac{\Delta B_\perp}{\sqrt{2}} & 0 & 0 & 0 & B & 0 \\ \frac{\Delta B_\perp}{\sqrt{2}} & 0 & 0 & 0 & 0 & -B \end{pmatrix}. \quad (13)$$

For simplicity, we may eliminate the states $|S(2,0)\rangle$ and $|T_-(1,1)\rangle$ from this equation, because they are very high in energy. We then perform free-induction simulations in the presence of charge noise, with the initial state $\rho(t = 0) = |i\rangle\langle i|$, where $|i\rangle = (|0\rangle + |1\rangle)/\sqrt{2}$ and $|0\rangle$ and $|1\rangle$ are the two low-energy qubit eigenstates. Averaging over noise realizations, as described in the main text, we obtain a final result for the density matrix $\rho(t)$. Fitting this to a relaxation equation of form

$$1 - |\rho_{T_+}(t)| = \frac{1}{4}\exp\left[-(t/T_1)\right] + \frac{3}{4} \quad (14)$$

where $\rho_{T_+}(t) = \langle T_+(1,1)|\,\rho(t)\,|T_+(1,1)\rangle$, yields the relaxation time T_1 for leakage to the $|T_+(1,1)\rangle$ state. In our simulations, we assume $\Delta B_\perp = \Delta B$, which gives the correct order of magnitude for $\Delta B_\perp$, although its exact value depends on the geometry of the nanomagnet[8].

In the second step of the procedure, we construct an appropriate qubit-cavity-qubit Hamiltonian H, in analogy with the main text, and solve the expanded master equation

$$\begin{aligned}\dot\rho = &-\frac{i}{\hbar}[H,\rho] + \frac{\kappa}{2}(2a\rho a^\dagger - a^\dagger a\rho - \rho a^\dagger a) \\ &+ \sum_{j=a,b}\Big[\frac{1}{2T_\varphi}(\sigma_{z,j}\rho\sigma_{z,j} - \rho) + \frac{1}{2T_L}(2\sigma_{L,j}\rho\sigma^\dagger_{L,j} - \sigma^\dagger_{L,j}\sigma_{L,j}\rho - \rho\sigma^\dagger_{L,j}\sigma_{L,j}) + \frac{1}{2T_1}(2\sigma_{L',j}\rho\sigma^\dagger_{L',j} - \sigma^\dagger_{L',j}\sigma_{L',j}\rho - \rho\sigma^\dagger_{L',j}\sigma_{L',j})\Big],\end{aligned}$$

which incorporates the expanded two-qubit basis set, and the leakage time T_1 obtained above. Here, we have defined $\sigma_{L',i} = \sum_{n=0,1}\sigma_{nT_+,i}$.

Supplementary Note 6. QUBIT OPERATION AT THE SOP

In the main text, we focused on the new TSS and ATSS working points proposed in this work. However, it is interesting to compare the performance of these gates to gates operated at the SOP. In this Supplementary Section, we perform simulations at the SOP with the same noise parameters used in our previous TSS/ATSS simulations.

At the SOP, we note that the transverse dipolar coupling is identically zero. It is still possible to perform one- and two-qubit gate operations, however, by applying an AC drive[9]. In ref 9, an AC drive was (theoretically) applied to the detuning parameter. However, at the SOP, the curvature coupling[10] associated with the detuning parameter, $\hbar|d^2\omega_q/d\varepsilon^2| \ll 1$, yielding a very slow gate. In the current calculation, we therefore manipulate the tunnel coupling[2], because its corresponding curvature coupling is much larger. Assuming $U = 3$ meV, a negligible magnetic field gradient ($\Delta B \approx 0$), and a typical experimental value for the singlet-triplet energy splitting[2] ($J/h = 160$ MHz), we evaluate Eq. (1) of the main text at the SOP ($\varepsilon = -U$) to obtain an estimate for the tunnel coupling: $\tau/h = 5.4$ GHz. We introduce time dependence into the tunnel-coupling parameter, $\Delta\tau(t)$, through the interaction Hamiltonian

$$H^{\tau}_{\text{int}} = \Delta\tau \begin{pmatrix} 0 & 0 & \sqrt{2} & \sqrt{2} \\ 0 & 0 & 0 & 0 \\ \sqrt{2} & 0 & 0 & 0 \\ \sqrt{2} & 0 & 0 & 0 \end{pmatrix}. \tag{15}$$

The time dependence of the detuning parameter is given by Eq. (2) of the main text.

To compute the coherence time T_2^* at the SOP, we follow the usual procedure for generating $1/f$ noise, as described in Methods. As in the main text, we consider detuning noise with amplitude of $c_\varepsilon = 0.56$ μeV, which is equivalent to $\sigma_\varepsilon = 2$ μeV. However, the effects of detuning noise are strongly suppressed at the SOP, yielding tunnel-coupling noise as the (probable) dominant noise source. We therefore consider fluctuations of both the detuning and tunnel-coupling parameters, $\delta\varepsilon(t)$ and $\delta\tau(t)$. Following ref 3 (see their Supplementary Materials), we assume $c_\tau \approx (d\tau/dE_b)c_\varepsilon \approx 3.2 \times 10^{-3} c_\varepsilon$ for the amplitudes of the $1/f$ noise distributions, where E_b is the height of the tunnel barrier in units of energy. For our simulations, we assume completely uncorrelated noise in the $\delta\varepsilon(t)$ and $\delta\tau(t)$ time series. In this way, we obtain a coherence time of $T_2^* = 5.94$ μs, which is dominated by tunnel-coupling noise, with negligible leakage outside the qubit subspace.

To characterize single-qubit gates, we assume it is possible to drive the tunnel coupling with an amplitude of one tenth of its average value, $\tau_{\text{AC}}/h = 540$ MHz, by applying an AC voltage signal to an appropriate top gate. The total tunnel-coupling dynamics is therefore described as $\Delta\tau(t) = \tau_{\text{AC}}\cos(\omega_d t) + \delta\tau(t)$, where we assume resonant driving with $\omega_d = \omega_q = J/\hbar$. Note that both leakage states, $|S(0,2)\rangle$ and $|S(2,0)\rangle$, play an important role in the dynamics near the SOP. The master equation, Eq. (9) of the main text, must therefore be extended, in the obvious way, to accommodate this 4D Hilbert space. We solve the resulting master equation, using the value of T_2^* obtained above and setting $T_L \to \infty$, obtaining a fidelity of $F = 99.6\%$ for an X_π rotation.

For two-qubit gates, we again consider tunnel-coupling driving, and we consider the dispersive limit for coupling to the resonator, since the qubit frequency ω_q is much smaller than the resonator frequency ω_r when $\Delta B \approx 0$. At the SOP, the native two-qubit gate is a CZ gate due to the dominant longitudinal coupling. (This is in contrast to the main text, where the native two-qubit gate for the TSS is iSWAP.) As suggested in ref 9, such gates can be implemented by driving both qubits at the same frequency, ω_d. The qubits may be coupled at opposite antinodes of the resonator, for example, in which case they should be driven 180° out of phase.

Following the derivation of the two-qubit coupling Hamiltonian in ref 9, but replacing the detuning driving with tunnel-coupling driving, we obtain the effective curvature Hamiltonian between one of the qubits and the resonator:

$$H^I_{\text{curv}} = \hbar\Delta a^\dagger a + \frac{\tau_{\text{AC}}}{4}\frac{d^2\omega_q}{d\tau^2}\bigg|_{\text{SOP}} g_0(a + a^\dagger)\sigma_z, \tag{16}$$

where $\Delta = \omega_r - \omega_d$. Here, we have removed excited qubit states from the derivation, since noise-induced leakage was found to be negligible. We have also averaged over rapidly oscillating terms and moved to an interaction picture with respect to $H_0 = \hbar\omega_d a^\dagger a + \frac{1}{2}\hbar\tilde{\omega}_q$, where $\hbar\tilde{\omega}_q = \hbar\omega_q + \frac{\hbar}{2}\frac{d^2\omega_q}{d\tau^2}\Big|_{\text{SOP}}(g_0^2 + \tau^2/2)$. [The superscript I in Eq. (16) indicates the interaction picture.] Including a second qubit now gives

$$H^I_{qqr} = \hbar\Delta a^\dagger a + \sum_{i=1,2}\frac{\tau_{\text{AC,i}}}{4}\frac{d^2\omega_{q,i}}{d\tau_i^2}\bigg|_{\text{SOP}} g_{0,i}(a + a^\dagger)\sigma_{z,i}. \tag{17}$$

Defining

$$g_i \equiv \frac{\tau_{\text{AC},i}}{2}\frac{d^2\omega_{q,i}}{d\tau_i^2}\bigg|_{\text{SOP}} g_{0,i}, \tag{18}$$

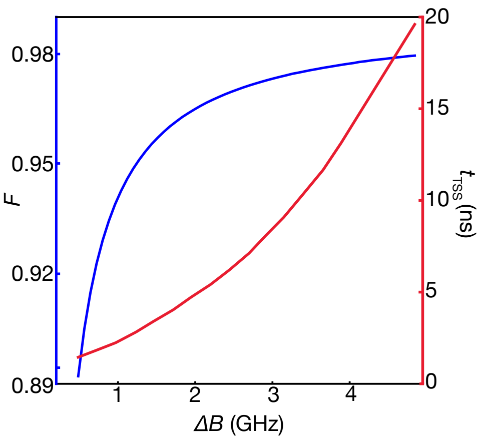

Supplementary Fig. 4. Fidelity (blue) and optimized ramp time for the TSS portion of the ramp, $t_{\rm TSS} = (\varepsilon_{\rm opt} - \varepsilon_{\rm crit})/v$ (red), as a function of ΔB, after optimizing the adiabatic ramp rate v. The adiabatic sweep fidelity follows a similar trend as the two-qubit gate fidelity: a monotonic increase with ΔB. In the first ramp step, from $\varepsilon = \varepsilon_{\rm opt}$ to $\varepsilon_{\rm crit}$, the ramp rate is mainly limited by leakage. In the second ramp step, from $\varepsilon = \varepsilon_{\rm crit}$ to the SOP, leakage and decoherence are negligible, so the ramp rate is not an important constraint.

and setting $g^{(1)} = g^{(2)} \equiv g$ for simplicity, we obtain the effective interaction

$$H^I_{qqr} = \hbar\Delta a^\dagger a + \frac{g}{2}(a + a^\dagger)(\sigma_{z,1} + \sigma_{z,2}), \tag{19}$$

which yields a CZ gate for the gate time $t_g = \pi\hbar\sqrt{n}/g$, when $\hbar\Delta = 2\sqrt{n}g$; here, n is the number of electrons in the resonator. Inserting H^I_{qqr} into master equation (9) of the main text, and using $T_\varphi = T_2^* = 5.94\ \mu$s, $\tau_{\rm AC,1} = \tau_{\rm AC,2} = 540$ MHz, and $n = 1$ yields a two-qubit gate fidelity of $F = 93.6\%$. We note that the current two-qubit gating protocol and operating points have not been optimized, and that higher-fidelity gates have been predicted[9].

We conclude that it is possible to perform single- and two-ST-qubit gates at the SOP using tunnel-coupling driving, without requiring a high magnetic field gradient. Single-qubit gates, in particular, can be achieved with high fidelities in this way. In comparison, the TSS provides potentially higher gate fidelities, but requires a high magnetic field gradient.

Supplementary Note 7. ADIABATIC TRANSITIONS BETWEEN TSS, ATSS, AND SOP OPERATING POINTS

The TSS, ATSS, and SOP working points offer different benefits and resources for qubit operation and idling. It is possible to transition between these operating points while continuously remaining at a sweet spot; however, fast transitions can cause leakage, while slow transitions can cause dephasing due to charge noise. In this Supplemental Section we optimize transition rates to determine the time scales for adiabatic operation.

Here, we consider a full transition from the TSS, to the ATSS, and finally to the SOP, all the while tuning ε and τ to remain at a sweet spot. While the optimal values of ε and τ are fixed, for a given value of ΔB, the transition rates are not. For simplicity, we consider a simple scenario involving linear ramps. If $\varepsilon_{\rm opt}$ corresponds to the optimal value in ε of Fig. 4(a) of the main text, for a given ΔB, and if $\varepsilon_{\rm crit}$ is the detuning value where the TSS and ATSS merge, then the distinct TSS and ATSS ramps are defined by

$$\text{TSS:} \begin{cases} \varepsilon(t) = \varepsilon_{\rm opt} - v_1\, t \\ \tau(t) = \tau_{\rm SS}(\varepsilon(t)) \end{cases}, \tag{20}$$

for $t \in [0, (\varepsilon_{\rm opt} - \varepsilon_{\rm crit})/v_1]$, and

$$\text{ATSS:} \begin{cases} \varepsilon(t) = \varepsilon_{\rm crit} - v_2\, t \\ \tau(t) = \tau_{\rm SS2}(\varepsilon(t)) \end{cases}, \tag{21}$$

for $t \in [(\varepsilon_{\rm opt} - \varepsilon_{\rm crit})/v_1, (\varepsilon_{\rm opt} + U)/v_2]$. Here, $\tau_{\rm SS2}(\varepsilon)$ describes the evolution of the ATSS sweet spot. Finally, we simplify the optimization procedure to comprise a single fitting parameter (v) by setting $v_1 = v_2 = v$.

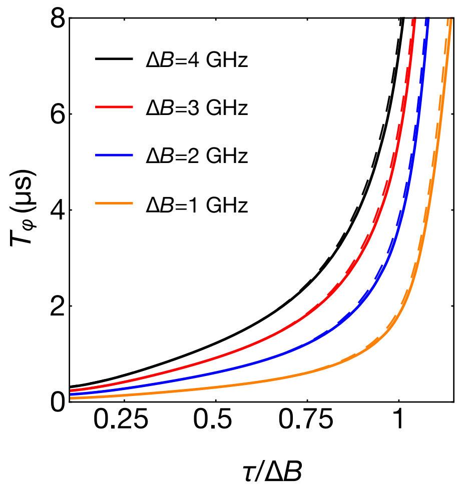

Supplementary Fig. 5. Dephasing time T_φ due to quasistatic (non-Markovian) detuning fluctuations (solid lines), or obtained from a Markovian master equation (dashed lines).

To estimate the fidelity of the transition, we solve the master equation of a single qubit coupled to a resonator, with four basis states (no polarized triplets). The initial state is taken to be the eigenstate $|1\rangle$ at the optimal TSS point, while the ideal final state is taken to be the corresponding eigenstate $|\tilde{1}\rangle$ at the SOP. At a given time, t, we adopt T_φ and T_L values consistent with $\varepsilon(t)$ and $\tau(t)$, as defined above. We then compute the fidelity for a given value of v,

$$F[v] = \mathrm{Tr}\left[\sqrt{\rho_{\mathrm{act}}\rho_{\mathrm{id}}}\,\right], \tag{22}$$

where ρ_{act} is the actual outcome of the master equation simulation, and $\rho_{\mathrm{id}} \equiv |\tilde{1}\rangle\langle\tilde{1}|$. Finally, we maximize F with respect to v, obtaining the results shown in Supplementary Fig. 4. The resulting fidelity is mainly limited by T_L in the first ramp, which goes through a minimum along the trajectory. In the second ramp step, both leakage and dephasing are weak, relaxing the speed constraint. Hence, the second ramp is only weakly affected by the ramp rate. We can therefore improve the fidelity by increasing ΔB, which in turn increases T_L, as per Fig. 3(c) of the main text. Alternatively, we note that there is room for improvement in the optimization procedure. A simple improvement would be to allow for independent ramp rates v_1 and v_2. More generally, we could allow for nontrivial, nonlinear ramps for both ε and τ. These changes would most likely provide faster ramps in the regime where dephasing and leakage are most harmful. It could also be beneficial to idle the qubit at the ATSS rather than the SOP, which would eliminate the second ramp step. Finally, we note that the procedure described above only optimizes an isolated ramp; by incorporating the ramp into a given gate protocol, for example a two-qubit gate operation, we would eliminate one of the ramp steps, which would improve the total fidelity.

Supplementary Note 8. MARKOVIAN TREATMENT OF DEPHASING NOISE

The dephasing parameter T_φ is determined by fluctuations that are slower than typical qubit time scales, which are therefore non-Markovian by definition. While such phenomena are commonly described by Markovian master equations[11], there is no guarantee that such an approach is accurate for any given problem.

In this section, we show that Markovian master equations provide a good description of dephasing in the problem we consider by demonstrating that independent Markovian and non-Markovian treatments of dephasing give nearly identical results. The first simulation describes non-dephasing effects (i.e., leakage) using the Markovian master Eq. (9) of the main text; however dephasing is introduced in non-Markovian fashion by adding a quasistatic shift to the detuning parameter, for many different shifts taken from a Gaussian distribution with a standard deviation $\sigma_\varepsilon = 2$ μeV. After averaging the resulting density matrices from many such simulations, we fit the exponential decay of the off-diagonal element of the density matrix, $\langle\rho_{01}\rangle$ to determine T_φ. In the second simulation, we introduce dephasing through the standard T_φ Lindblad term in the master equation, using the value of T_φ obtained above. This simulation is just performed once, and T_φ is recomputed by fitting the exponential decay of the off-diagonal matrix

element. These two simulations differ only in their treatment of dephasing. The results of the simulations are plotted in Supplementary Fig. 5, showing nearly identical behavior. This demonstrates that the Markovian master equation robustly describes dephasing physics in the ST system considered here.

---

[1] Wolfram Research, Inc., "Mathematica, Version 11.2," Champaign, IL, 2017.
[2] M. D. Reed, B. M. Maune, R. W. Andrews, M. G. Borselli, K. Eng, M. P. Jura, A. A. Kiselev, T. D. Ladd, S. T. Merkel, I. Milosavljevic, E. J. Pritchett, M. T. Rakher, R. S. Ross, A. E. Schmitz, A. Smith, J. A. Wright, M. F. Gyure, and A. T. Hunter, Phys. Rev. Lett. **116**, 110402 (2016).
[3] P. Huang, N. M. Zimmerman, and G. W. Bryant, npj Quantum Inform. **4**, 62 (2018).
[4] F. Yan, S. Gustavsson, J. Bylander, X. Jin, F. Yoshihara, D. G. Cory, Y. Nakamura, T. P. Orlando, and W. D. Oliver, . **4**, 2337 (2013).
[5] E. Kawakami, T. Jullien, P. Scarlino, D. R. Ward, D. E. Savage, M. G. Lagally, V. V. Dobrovitski, M. Friesen, S. N. Coppersmith, M. A. Eriksson, and L. M. K. Vandersypen, Proceedings of the National Academy of Sciences **113**, 11738 (2016), http://www.pnas.org/content/113/42/11738.full.pdf.
[6] L. V. Assali, H. M. Petrilli, R. B. Capaz, B. Koiller, X. Hu, and S. D. Sarma, Phys. Rev. B **83**, 165301 (2011).
[7] A. Blais, R.-S. Huang, A. Wallraff, S. M. Girvin, and R. J. Schoelkopf, Phys. Rev. A **69**, 062320 (2004).
[8] J. Yoneda, T. Otsuka, T. Takakura, M. Pioro-Ladrière, R. Brunner, H. Lu, T. Nakajima, T. Obata, A. Noiri, C. J. Palmstrøm, A. C. Gossard, and S. Tarucha, Appl. Phys. Express **8**, 084401 (2015).
[9] S. P. Harvey, C. G. L. Bøttcher, L. A. Orona, S. D. Bartlett, A. C. Doherty, and A. Yacoby, Phys. Rev. B **97**, 235409 (2018).
[10] R. Ruskov and C. Tahan, Phys. Rev. B **99**, 245306 (2019).
[11] V. Srinivasa, J. M. Taylor, and C. Tahan, Phys. Rev. B **94**, 205421 (2016).
