# Supplementary material for: Enhancing the dipolar coupling of a S-T0 qubit with a transverse sweet spot
Source: Nat Commun. 2019 Dec 10;10:5641. doi: 10.1038/s41467-019-13548-w (PMC6904552; doi:10.1038/s41467-019-13548-w)
Supplement: Supplementary file 2 — Description of Additional Supplementary Files [file 41467_2019_13548_MOESM2_ESM.pdf]

Description of Additional Supplementary Files

File Name: Supplementary Software 1
Description: Mathematica notebook for the characterization of the SOP
